# Identifying genetic variants associated with the ICD10 (International Classification of Diseases10)-based diagnosis of cerebrovascular disease using a large-scale biomedical database

**DOI:** 10.1371/journal.pone.0273217

**Published:** 2022-08-22

**Authors:** Fahad Alkhalfan, Alex Gyftopoulos, Yi-Ju Chen, Charles H. Williams, James A. Perry, Charles C. Hong

**Affiliations:** University of Maryland School of Medicine, Baltimore, Maryland, United States of America; Ohio State University, UNITED STATES

## Abstract

**Objectives:**

To utilize the UK Biobank to identify genetic variants associated with the ICD10 (International Classification of Diseases10)-based diagnosis of cerebrovascular disease (CeVD).

**Background:**

Cerebrovascular disease occurs because of a complex interplay between vascular, environmental, and genetic factors. It is the second leading cause of disability worldwide. Understanding who may be genetically predisposed to cerebrovascular disease can help guide preventative efforts. Moreover, there is considerable interest in the use of real-world data, such as EHR (electronic health records) to better understand disease mechanisms and to discover new treatment strategies, but whether ICD10-based diagnosis can be used to study CeVD genetics is unknown.

**Methods:**

Using the UK Biobank, we conducted a genome-wide association study (GWAS) where we analyzed the genomes of 11,155 cases and 122,705 controls who were sex, age and ancestry-matched in a 1:11 case: control design. Genetic variants were identified by Plink’s firth logistic regression and assessed for association with the ICD10 codes corresponding to CeVD.

**Results:**

We identified two groups of SNPs closely linked to *PITX2* and *LRRTM4* that were significantly associated with CeVD in this study (p < 5 x 10^−8^) and had a minor allele frequency of > 0.5%.

**Discussion:**

Disease assignment based on ICD10 codes may underestimate prevalence; however, for CeVD, this does not appear to be the case. Compared to the age- and sex-matched control population, individuals with CeVD were more frequently diagnosed with comorbid conditions, such as hypertension, hyperlipidemia & atrial fibrillation or flutter, confirming their contribution to CeVD. The UK Biobank based ICD10 study identified 2 groups of variants that were associated with CeVD. The association between *PITX2* and CeVD is likely explained by the increased rates of atrial fibrillation and flutter. While the mechanism explaining the relationship between *LRRTM4* and CeVD is unclear, this has been documented in previous studies.

## Introduction

Cerebrovascular disease (CeVD), which commonly manifests as a stroke, is one of the leading causes of serious long-term disability and the second leading cause of death worldwide [1]. Although there is a decline in cerebrovascular disease mortality in the United States, cerebrovascular disease continues to be the fourth leading cause of death among women and fifth leading cause of death among men, with an estimated 795,000 new or recurrent strokes occurring each year [2]. What is even more concerning is that there has been a reported 19% increase in the rate of cerebrovascular disease in women between the ages of 35 and 64 [1]. Cerebrovascular disease is the result of a complex interplay between vascular, genetic and environmental factors [3]. Recent studies have demonstrated that traditional risk factors such as diabetes, hyperlipidemia, hypertension, and smoking may only account for a small proportion of variance in atherosclerosis, suggesting that there may be novel non-traditional and genetic risk factors contributing to this process that have yet to be identified [3].

There is considerable interest in the use of real-world data, such as EHR (electronic health records) to better understand disease mechanisms and to discover new treatment strategies [4, 5]. The UK Biobank is a large, ongoing prospective cohort study that recruited 502,682 UK participants between 2006–2010. UK Biobank has compiled extensive health-related records and genetic data from participants [6, 7]. In this manuscript, using ICD-10 diagnostic codes as a diagnosis of CeVD, we conducted a genome-wide association study (GWAS) to identify genetic variants associated with cerebrovascular disease.

## Methods

### Ethical approval

The current study involved de-identified data obtained from the UK Biobank Resource under Application Number 49852. It has received the proper ethical oversight, including the determination by the University of Maryland, Baltimore Institutional Review Board that the study is not human research (IRB #: HF-00088022).

### Study population

We carried out a GWAS using the UK Biobank to assess for statistically significant single nucleotide polymorphisms (SNPs) and the clinical manifestations of cerebrovascular disease at the population level. The UK Biobank recruited 502,682 UK participants between 2006 and 2010. The participants were between the ages of 40 and 69 at the time of recruitment. Extensive health-records were collected from participants, including clinical and genetic data with over 820,000 genotyped SNPs and up to 90 million imputed variants available for most individuals [7].

### Genome-wide association study

Using data from the UK Biobank Resource on 487,310 subjects with imputed genotypes, we performed quality control by removing those with genetic relatedness exclusions (Data-Field 22018—UKB, https://biobank.ctsu.ox.ac.uk/crystal/field.cgi?id=22018; 1532 subjects), sex chromosome aneuploidy (Data-Field 22019 –UKB, https://biobank.ctsu.ox.ac.uk/crystal/field.cgi?id=22019; 651 subjects), mismatch between self-reported sex and genetically determined sex (Data-Field 31 –UKB, https://biobank.ctsu.ox.ac.uk/crystal/field.cgi?id=31; Data-Field 22001 –UKB, https://biobank.ctsu.ox.ac.uk/crystal/field.cgi?id=22001; 372 subjects), recommended genomic analysis exclusions (Data-Field 22010—UKB, https://biobank.ctsu.ox.ac.uk/crystal/field.cgi?id=22010; 480 subjects), and outliers for heterozygosity or missing rate (Data-Field 22017 –UKB, https://biobank.ctsu.ox.ac.uk/crystal/field.cgi?id=22077; 968 subjects).

Cases were defined using the International Standard Classification of Diseases and Related Health Problems, 10^th^ edition (ICD-10) diagnostic codes related to cerebrovascular disease as primary or secondary diagnosis at the time of this analysis (August 2021). **Table 1** has a list of all ICD-10 codes included as cases in this analysis. The set of selected cases was purged of relatedness by removing one from each related pair in an iterative fashion until no subjects remained. Relatedness was defined as a kinship coefficient greater than 0.44. This treats third-degree relationships, which was defined a kinship coefficient of 0.0625, as related. Kinship coefficients for all subject pairs greater than 0.44 were provided by the UK Biobank as part of the standard set. To select the controls, all cases and related individuals were first removed from the pool. From the reduced pool, we were able to select 11 control subjects for each case, matching for age, sex and ancestry using incremental tolerances. The tolerance for age ranged from 0 (exact match) up to 7 years. Ancestry matching was performed with principal components (PCs) supplied by the UK Biobank. The mathematical distance in a graph plotting the PC1 x PC2 was used to test similarity in ancestry. The “distance” in ancestry tolerance ranged from 2 PC units to a maximum of 80 PC units with PC1 (**S1 Fig**), ranging from 0 to +400 and PC2 ranging from -300 to +100 units. These tolerances were used to identify the 11 matching controls for every case.

**Table 1:** List of ICD-10 codes included as cerebrovascular disease cases.

ICD-10 code	Diagnosis
**G45.0**	Vertebro-basilar artery syndrome
**G45.1**	Carotid artery syndrome (hemispheric)
**G45.2**	Multiple and bilateral precerebral artery syndromes
**G45.3**	Amaurosis fugax
**G45.4**	Transient global amnesia
**G45.8**	Other transient cerebral ischemic attacks and related syndromes
**G45.9**	Transient cerebral ischemic attack
**G46.0**	Middle cerebral artery syndrome
**G46.1**	Anterior cerebral artery syndrome
**G46.2**	Posterior cerebral artery syndrome
**G46.3**	Brain stem stroke syndrome
**G46.4**	Cerebellar stroke syndrome
**G46.5**	Pure motor lacunar syndrome
**G46.6**	Pure sensory lacunar syndrome
**G46.7**	Other lacunar syndromes
**G46.8**	Other vascular syndromes of brain in cerebrovascular diseases
**I63.0**	Cerebral infarction due to thrombosis of precerebral arteries
**I63.1**	Cerebral infarction due to embolism of precerebral arteries
**I63.2**	Cerebral infarction due to unspecified occlusion or stenosis of precerebral arteries
**I63.3**	Cerebral infarction due to thrombosis of cerebral arteries
**I63.4**	Cerebral infarction due to embolism of cerebral arteries
**I63.5**	Cerebral infarction due to unspecified occlusion or stenosis of cerebral arteries
**I63.6**	Cerebral infarction due to cerebral venous thrombosis, nonpyogenic
**I63.8**	Other cerebral infarction
**I63.9**	Cerebral infarction, unspecified
**I64**	Stroke, not specified as hemorrhage or infarction
**I65.0**	Occlusion and stenosis of vertebral artery
**I65.1**	Occlusion and stenosis of basilar artery
**I65.2**	Occlusion and stenosis of carotid artery
**I65.3**	Occlusion and stenosis of multiple and bilateral precerebral arteries
**I65.8**	Occlusion and stenosis of other precerebral artery
**I65.9**	Occlusion and stenosis of unspecified precerebral artery
**I66.0**	Occlusion and stenosis of middle cerebral artery
**I66.1**	Occlusion and stenosis of anterior cerebral artery
**I66.2**	Occlusion and stenosis of posterior cerebral artery
**I66.3**	Occlusion and stenosis of cerebellar arteries
**I66.4**	Occlusion and stenosis of multiple and bilateral cerebral arteries
**I66.8**	Occlusion and stenosis of other cerebral artery
**I66.9**	Occlusion and stenosis of unspecified cerebral artery
**I67.2**	Cerebral atherosclerosis
**I67.9**	Cerebrovascular disease, unspecified
**I69.3**	Sequelae of cerebral infarction
**I69.4**	Sequelae of stroke, not specified as hemorrhage or infarction
**I69.8**	Sequelae of other and unspecified cerebrovascular diseases

The association analysis was carried out using Plink’s firth logistic regression model and adjusted for age, sex and the 5 PCs using data supplied by the UK Biobank Resource [8]. Firth regression was chosen because it has been shown to provide the best combination of control for type 1 error and power for detection of low frequency variants [9, 10]. The cases of CeVD were analyzed with the 40 million imputed genetic variants provided by the UK Biobank with imputation quality scores of greater than 0.70. The covariates included in the analyses were sex, age and principal components 1 through 5. Although pre-calculated PC for the first 40 principal components was supplied by the UK Biobank, our preliminary analysis showed that only the first 5 PCs were considered significant (p value less than 0.05). Therefore, only the first 5 PCs were included in this GWAS.

### Identification of variants for the CeVD phenotype

Variants were determined to be of interest if they had a minor allele frequency (MAF) of 0.5% or greater and met the standard threshold for genome-wide significance (p-value of less than 5 x 10^−8^). Identified variants were assessed for the presence of previously reported phenotypic associations using pheweb.org and the Cerebrovascular Disease Knowledge Portal. Pheweb.org is a GWAS dataset for electronic health record-derived disease associations from the white British participants in the UK Biobank. PheWeb utilizes a generalized mixed model association test that uses the saddle point approximation to account for case-control imbalance and imputed using the Haplotype Reference Consortium Panel [11]. The Cerebrovascular Disease Knowledge Portal (cd.hugeamp.org) is an online platform that provides comprehensive quality-assured genetic and phenotypic data on a large number of patients with stroke worldwide. It includes data from up to 18 different datasets [12].

## Results

A total of 11,155 individuals (6,777 males and 4,378 females) were identified as CeVD cases at the time of this analysis (January 2021). This represents a prevalence of 22.2 cases per 1,000 people in the study population. Around 60% of the cases were male. The average age of the cases at the time of diagnosis was 63.8 years (+/- 8.3). Additionally, cases tend to have a higher BMI (28.5 vs 27.5, p <0.001) and a greater waist circumference (37.6 inches vs 36.4 inches, p <0.001). Most cases described their ethnic background as British (89.6%) with a smaller proportion reporting their ethnic background as Irish (3.0%) and Indian (1.0%). Demographic characteristics are shown in **Table 2**. Relevant biometric and biochemical profiles of both the cases and controls can be seen in **Table 3**. A more expansive list can be seen in **S1 Table**.

**Table 2:** Demographic characteristics of individuals with cerebrovascular disease and their respective controls.

	Cases	Controls
**Cases (#)**	11,155	122,705
**Male**	60.8%	60.8%
**Males, mean age at time of diagnosis (years ± SD)**	63.76 ± 8.18	Age matched to cases
**Females, mean age at time of diagnosis (years ± SD)**	63.86 ± 8.52	Age matched to cases
**Ethnic Background**		
** White British**	89.6%	89.8%
** White Irish**	3.0%	2.5%
**Other white** **background**	2.5%	2.9%
** Indian**	1.0%	1.2%
** Pakistani**	0.4%	0.4%
** African**	0.6%	0.6%
** Caribbean**	0.9%	0.9%

**Table 3:** Relevant biometrics and biomarkers of cases and controls.

Biometric/Biomarker	Cases (N = 11,155)	Controls (N = 122,705)	P Value	Clinical Significance
**BMI (kg/m^2^)**	28.5	27.5	<0.001	N
**Waist Circumference (in)**	37.6	36.4	<0.001	N
**Weight (lbs)**	180	176	<0.001	N
**Systolic blood pressure (mmHg)**	145	144	<0.001	N
**Diastolic blood pressure (mmHg)**	83.0	82.9	NS	N
**Mean arterial blood pressure (mmHg)**	103	103	<0.001	N
**Pulse Pressure (mmHg)**	60.3	59.1	<0.001	N
**Pulse Rate (bpm)**	70.6	69.3	<0.001	N
**Apolipoprotein A (mg/dL)**	148	153	<0.001	N
**Apolipoprotein B (mg/dL)**	96.7	103	<0.001	N
**Total Cholesterol (mg/dL)**	202	218	<0.001	M
**C Reactive Protein (mg/dL)**	99.5	74.4	<0.001	M
**HDL (mg/dL)**	51.8	54.9	<0.001	N
**LDL (mg/dL)**	125	137	<0.001	M
**Lipoprotein A (mg/dL)**	22.8	21.1	<0.001	N
**Triglycerides (mg/dL)**	167	161	<0.001	N
**Glucose (mg/dL)**	98.3	93.4	<0.001	N
**Hemoglobin A1c (%)**	5.7	5.5	<0.001	N
**Hemoglobin (g/dL)**	14.3	14.4	<0.001	N
**Hematocrit (percent)**	41.5	41.8	<0.001	N
**Mean Corpuscular Volume (fL)**	91.8	91.4	<0.001	N
**White Blood Cell Count (1x 10^9^/L)**	7.4	6.9	<0.001	N
**Platelet Count (1 x 10^9^/L)**	251	246	<0.001	N
**Blood Urea Nitrogen (mg/dL)**	35.2	33.8	<0.001	N
**Creatinine (mg/dL)**	0.79	0.75	<0.001	N
**Cystatin C (mg/L)**	1.02	0.94	<0.001	N
**Phosphate (mg/dl)**	3.56	3.56	<0.05	N
**Uric Acid (mg/dL)**	5.66	5.46	<0.001	N
**Microalbumin–Urine (mg/L)**	59.6	31.0	<0.001	M
**Creatinine–Urine (mmol/L)**	9710	9150	<0.001	L

NS: Not significant, p >0.05; N: No clinical significance; L: Low clinical significance; M: Medium clinical significance; H: High clinical significance

Relevant ICD-10 diagnoses associated with CeVD can be seen in **Table 4**. A full list of ICD-10 diagnoses associated with CeVD can be seen in **S2 Table**. Cases were more likely to be diagnosed with chronic renal failure (N18.9: 6% vs 1%, p <0.001) and acute renal failure (N17.9: 10% vs 2%, p <0.001) and had higher levels of urea (35.2 mg/dl vs 33.8 mg/dl, p <0.001) and creatinine (0.79 mg/dl vs 0.75 mg/dl, p <0.001) which is consistent with the increased rate of renal disease in the cases. Additionally, cases had higher rates of chronic ischemic heart disease (I25.9: 18% vs 5%, p <0.001), atherosclerotic heart disease (I25.1: 18% vs 7%, p <0.001), old myocardial infarction (I25.2: 12% vs 3%, p <0.001), left ventricular failure (I50.1: 6% vs 1%, p <0.001), atrial fibrillation and flutter (I48: 23% vs 6%, p <0.001), syncope and collapse (R55: 11% vs 3%, p <0.001), epilepsy (G40.9: 6% vs 1%, p <0.001) and personal use of long-term anticoagulants (Z92.1: 20% vs 4%, p <0.001). Cases were also more likely to have risk factors that are typically associated with cerebrovascular disease including tobacco use (Z72.0: 11% vs 3%, p <0.001), hyperlipidemia (E78.5: 8% vs 2%, p <0.001), primary hypercholesterolemia (E78.0: 41% vs 12%, p <0.001) and essential hypertension (I10: 66% vs 28%, p <0.001). Although the systolic blood pressure was higher in cases (145 mm Hg vs 144 mm Hg, p <0.001), there was no significant difference in the diastolic blood pressure (83 mm Hg in cases vs 82.9 in controls, p > 0.05). Finally, cases had a higher rate of type 2 diabetes mellitus (20% vs 7%, p <0.001) which could also be seen through cases having a higher hemoglobin A1c level (5.7% vs 5.5%, p <0.001) and higher serum glucose levels (98.3 mg/dl vs 93.4 mg/dl, p <0.001).

**Table 4:** Relevant ICD10 diagnoses associated with cerebrovascular disease cases.

ICD10 Diagnosis	Cases (N = 11,155)	Controls (N = 122,705)	P Value
**Peripheral vascular disease; unspecified (I73.9)**	6%	1%	<0.001
**Unstable angina (I20.0)**	6%	2%	<0.001
**Presence of aortocoronary bypass graft (Z95.1)**	7%	2%	<0.001
**Other forms of chronic ischemic heart disease (I25.8)**	9%	2%	<0.001
**Presence of coronary angioplasty implant and graft (Z95.5)**	7%	3%	<0.001
**Old myocardial infarction (I25.2)**	12%	3%	<0.001
**Chronic ischemic heart disease; unspecified (I25.9)**	18%	5%	<0.001
**Angina pectoris; unspecified (I20.9)**	17%	6%	<0.001
**Atherosclerotic heart disease (I25.1)**	18%	7%	<0.001
**Left ventricular failure (I50.1)**	6%	1%	<0.001
**Disorientation; unspecified (R41.0)**	6%	1%	<0.001
**Tendency to fall; not elsewhere classified (R29.6)**	6%	1%	<0.001
**Chronic renal failure (N18.9)**	6%	1%	<0.001
**Acute renal failure; unspecified (N17.9)**	10%	2%	<0.001
**Malaise and fatigue (R53)**	5%	1%	<0.001
**Epilepsy; unspecified (G40.9)**	6%	1%	<0.001
**Hyperlipidemia; unspecified (E78.5)**	8%	2%	<0.001
**Pleural effusion; not elsewhere classified (J90)**	6%	1%	<0.001
**Dizziness and giddiness (R42)**	7%	1%	<0.001
**Lobar pneumonia; unspecified (J18.1)**	8%	2%	<0.001
**Dyspnea (R06.0)**	7%	2%	<0.001
**Personal history of diseases of the nervous system and sense organs (Z86.6)**	26%	2%	<0.001
**Family history of ischemic heart disease and other diseases of the circulatory system (Z82.4)**	11%	4%	<0.001
**Iron deficiency anemia; unspecified (D50.9)**	5%	2%	<0.001
**Chronic obstructive pulmonary disease; unspecified (J44.9)**	10%	3%	<0.001
**Headache (R51)**	9%	2%	<0.001
**Dysphagia (R13)**	6%	2%	<0.001
**Tobacco use (Z72.0)**	11%	3%	<0.001
**Other chest pain (R07.3)**	7%	2%	<0.001
**Other specified abnormal findings of blood chemistry (R79.8)**	7%	3%	<0.001
**Alcohol use (Z72.1)**	7%	2%	<0.001
**Retention of urine (R33)**	7%	3%	<0.001
**Syncope and collapse (R55)**	11%	3%	<0.001
**Personal history of long-term (current) use of anticoagulants (Z92.1)**	20%	4%	<0.001
**Harmful use (F17.1)**	11%	3%	<0.001
**Anemia; unspecified (D64.9)**	10%	3%	<0.001
**Nausea and vomiting (R11)**	10%	3%	<0.001
**Depressive episode; unspecified (F32.9)**	11%	3%	<0.001
**Obesity; unspecified (E66.9)**	8%	4%	<0.001
**Personal history of diseases of the circulatory system (Z86.7)**	47%	4%	<0.001
**Atrial fibrillation and flutter (I48)**	23%	6%	<0.001
**Unknown and unspecified causes of morbidity (R69)**	8%	4%	<0.001
**Hypothyroidism; unspecified (E03.9)**	8%	4%	<0.001
**Chest pain; unspecified (R07.4)**	17%	7%	<0.001
**Non-insulin-dependent DM Without complications (E11.9)**	20%	7%	<0.001
**Pure hypercholesterolemia (E78.0)**	41%	12%	<0.001
**Essential (primary) hypertension (I10)**	66%	28%	<0.001

While cases were more likely to be diagnosed with hyperlipidemia and primary hypercholesterolemia, they had lower low-density lipoprotein (LDL) (125 mg/dl vs 137 mg/dl, p <0.001) and cholesterol levels (202 mg/dl vs 218 mg/dl, p <0.001) but higher triglycerides (167 mg/dl vs 161 mg/dl, p <0.001). Additionally, high-density lipoprotein (HDL) levels were lower in the cases (51.8 mg/dl vs 54.9 mg/dl, p <0.001). Interestingly, cases also had higher levels of C-reactive protein (CRP) (99.5 mg/dl vs 74.4 mg/dl, p <0.001).

**Fig 1:**
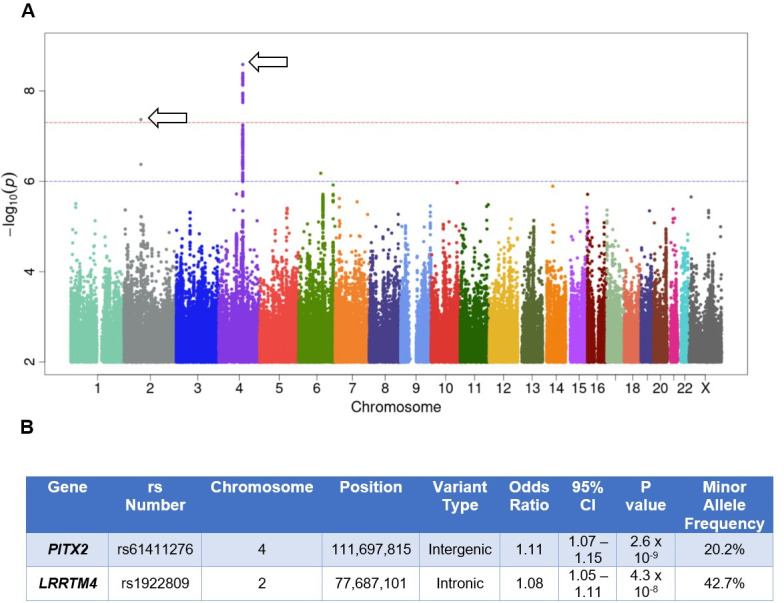
Genome-wide association study analysis for variants associated with cerebrovascular disease in the UK Biobank. **(A)** Manhattan plot of GWAS results (MAF > 0.5%) for CeVD identified 2 groups of SNPs that were significantly associated with CeVD **(B)** SNPs with the highest statistical significance from each of the two groups associated with CeVD. For the Manhattan plot, significance is displayed on the y-axis as -log_10_ of the *p*-value, with results ordered along the x-axis by chromosome (each colored bar represents a different chromosome). Arrows represents SNP associated with a genome-wide significance of 5 x 10^−8^. OR: Odds ratio; *PITX2*: Paired like homeodomain 2; *LRRTM4*: leucine rich repeat transmembrane neuronal 4; CI: Confidence Interval.

**Fig 2:**
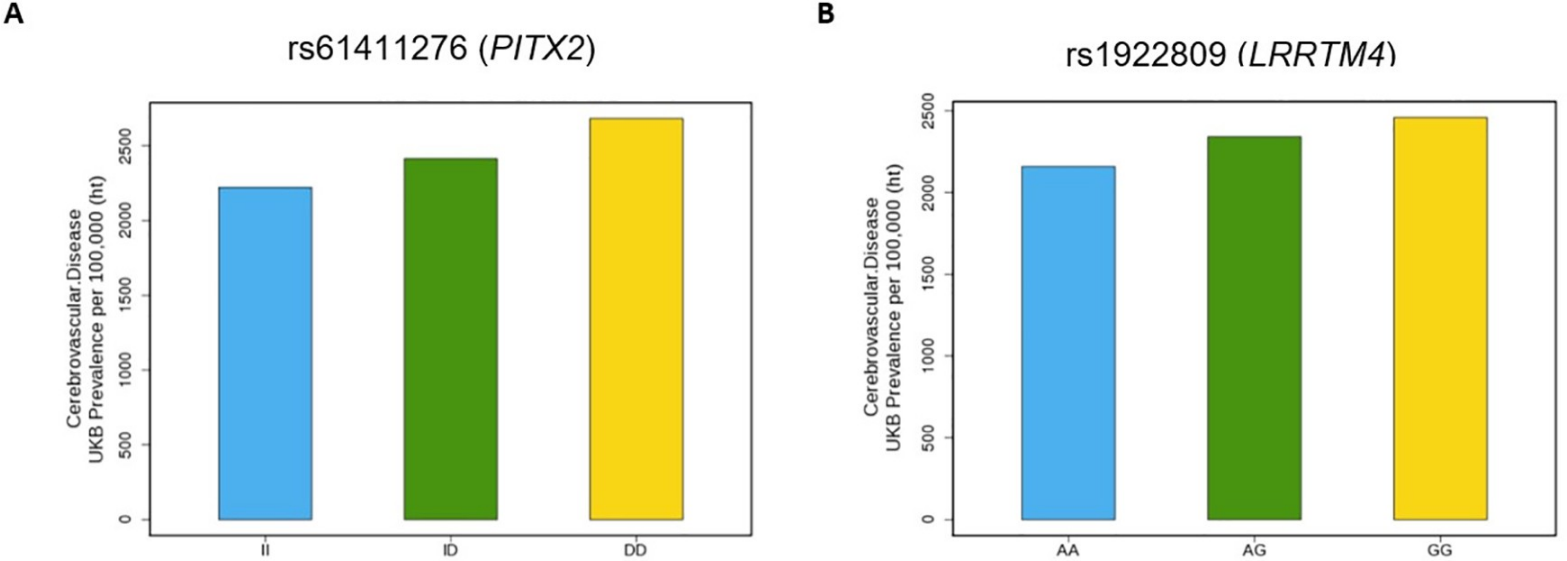
Single nucleotide polymorphisms associated with cerebrovascular disease in the UK Biobank. (A) Prevalence of CeVD increases with PITX2 variant rs61411276 status (2,222 per 100,000 for wild type; 2,414 per 100,000 for heterozygotes; 2,682 per 100,000 for homozygotes; p-value 2.6 x 10^−9^; OR 1.1 per D allele). (B) Prevalence of CeVD increases with LRRTM4 variant rs1922809 status (2,159 per 100,000 for wild type; 2,341 per 100,000 for heterozygotes; 2,458 per 100,000 for homozygotes; p-value 4.3 x 10^−8^; OR 1.08 per G allele). OR: Odds ratio; PITX2: Paired like homeodomain 2; LRRTM4: leucine rich repeat transmembrane neuronal 4.

GWAS identified two group of SNPs with a MAF of ≥ 0.5% that were associated with cerebrovascular disease with a p-value of less than 5 x 10^−8^ (**Figs 1 and 2)**. First, a group of 23 SNPS in close to proximity to sequences encoding *PITX2* (Paired like homeodomain 2, a gene that encodes the RIEG/PITX homeobox family) were found to be associated with CeVD (**S3 Table**). Of this group, the intergenic SNP rs61411276 (mean allele frequency of 20.2%) was found to be most significantly associated with the outcome, with an odds ratio (OR) of 1.11, 95% confidence interval of 1.07 to 1.15, and a p-value of 2.59 x 10^−9^. A second SNP (rs1922809) in close proximity to sequences encoding *LRRTM4* (leucine rich repeat transmembrane neuronal 4) was identified. The intronic SNP rs1922809 had an OR of 1.08, 95% confidence interval of 1.05 to 1.11, and a p-value of 4.29 x 10^−8^. Data from the PheWeb database was available for rs1922809 and showed that this SNP was associated with cerebrovascular disease. Data from the Cerebrovascular Disease Knowledge Portal was available for both SNPs. While the association between rs61411276 and ischemic stroke was not significant (OR = 1.04, p = 0.09327 with an effective sample size of 93,661), there was a significant association between rs1922809 and ischemic stroke (OR = 1.04, p = 0.0284 with an effective sample size of 513,323) and brain microbleeds (mixed or strictly deep, OR = 1.09, p = 0.0318 with an effective sample size of 4,848).

## Discussion

Through this ICD-10 based study, we identified two groups of SNPs near *PITX2* and *LRRTM4* that were significantly associated with CeVD. There has been a growing body of literature to suggest that although traditional risk factors such as smoking, diabetes and hypertension do increase the risk of CeVD, CeVD also has a genetic component that has yet to be fully understood [3]. With that in mind, although we matched for age, sex and ancestry, the cases in our study did have higher rates of traditional risk factors including smoking, diabetes mellitus, atrial fibrillation and flutter, hypertension and were more likely to have other atherosclerotic conditions such as atherosclerotic heart disease and peripheral vascular disease.

As with all GWAS studies, although we were able to demonstrate a statistically significant association between the novel loci identified in this manuscript and the development of CeVD, this does not imply causation. However, the findings of this study do have a biological plausibility that can be explained. For instance, the *PITX2* gene encodes a protein that regulates right-left differentiation of the embryonic heart and expressed in the adult left atrium. A deficiency in *PITX2* can result in electrical and structural remodeling in murine models and this may predispose patients to atrial fibrillation, a known risk factor for thromboembolic stroke [13]. Additionally, our findings are consistent with other studies that have described the association between *PITX2* and cardioembolic stroke [14]. In our analysis, cases had a significantly higher rate of atrial fibrillation and flutter (I48) and were more likely to be on anticoagulation as compared to cases. Interestingly, in both the cases and controls, the proportion of participants with atrial fibrillation increases with progression from wild-type to homozygotes for rs61411276 (Cases: II: 20%, ID: 26%, DD: 36%, p <0.001; Controls: II: 5%, ID: 7%, DD: 10%, p <0.001). However, there was no significant difference in the rate of atherosclerotic heart disease (I25.1) when comparing wild-type, heterozygotes and homozygotes in both cases and controls (p > 0.05). This would suggest that the increased rate of CeVD with rs61411276 is likely secondary to atrial fibrillation/flutter as opposed to atherosclerosis.

Also, the SNP rs1922809 closely linked to *LRRTM4* was associated with an increased risk of CeVD in our study. While the mechanism that can explain this association is not clear, this finding is consistent with the Cerebrovascular Disease Knowledge Portal. As seen in the Cerebrovascular Disease Knowledge Portal, rs1922809 was significantly associated with ischemic strokes and microbleeds in populations other than the UK Biobank [12]. *LRRTM4* is expressed in the central nervous system and the structure and expression profile of LRRTM4 mRNAs suggest that it may have a role and maintenance of the nervous system [15]. Additionally, it has been identified as a possible marker of cognitive impairment in one study and has been shown to play a role in synapse function [16, 17]. We performed a post-hoc power calculation on the signal for rs1922809 to assess the probability of detecting the OR that we found in our analysis. Using the power calculator on the University of Michigan website [18] we determined that we had 80% power to detect an OR of 1.10 and 30% power to detect an OR of 1.08.

There are limitations to this study that need to be highlighted. Firstly, although we were able to demonstrate a statistically significant association, this does not imply causality and the findings of the study will need to be verified in future analyses, ideally in a more diverse patient population. Due to the high prevalence of traditional risk factors associated with CeVD, it was not feasible to only include patients without traditional risk factors as this would have significantly reduced the power of this analysis. Additionally, as compared to a study that is dedicated specifically to studying CeVD, this analysis utilized data from the UK Biobank and identified cases using ICD10 codes. ICD10 based studies are limited by the fact that many diseases and medical diagnoses are often underdiagnosed. However, that does not seem to be the case here as the prevalence of cerebrovascular disease in our study group is similar to prevalences reported in recent literature [19]. In this study, controls are selected randomly from a pool of those who do not carry the ICD10 diagnosis, not those in whom CeVD has been specifically ruled out. However, ICD10 based studies also offer the opportunity to study a wide array of diseases and medical conditions, both common and rare, using an already available data source. One method of improving the accuracy of successfully differentiating between cases and controls is to incorporate imaging data, such as magnetic resonance imaging or angiography, into the analysis. Although this may limit the number of patients included in the study, it would more accurately distinguish between cases and controls. Ultimately, ICD10 based studies can help direct research to potential variants that were associated with CeVD so that future studies can then be designed to further explore and validate these findings in other cohorts and if valid, potentially be incorporated into a genetic risk score to better identify high risk patients.

## Conclusions

In this ICD-10 based study, we have identified two groups of SNPs (*PITX2* and *LRRTM4*) that were significantly associated with the diagnosis of CeVD in the UK Biobank (p < 5 x 10^−8^). The SNP (rs61411276) closely linked to *PITX2* gene was associated with an increased risk of atrial fibrillation and flutter which can explain the association between this gene and the increased CeVD risk. Although the mechanism is unclear, *LRRTM4* gene has been associated with cerebrovascular disease in past studies. While ICD10 based studies do have their limitations, they provide the opportunity to study a wide array of diseases and identify potential variants associated with medical conditions (both rare and common) that can then be validated and explored in further studies.

## Supporting information

S1 Table Full biometrics and biomarkers of cases and controls. (DOCX)

S2 Table ICD10 codes associated with cerebrovascular disease in the UK Biobank. (DOCX)

S3 Table Single nucleotide polymorphisms associated with cerebrovascular disease in this genome-wise association study. (DOCX)

S1 Fig Principal components (PCs) by ethnicity for UK Biobank participants. When selecting controls for comparison with cases, control subjects were selected from subjects within 80 units on the PC1 vs. PC2 graph. The size of 80 units is illustrated with the red boxes around subjects who are primarily European, Chinese, or African Ethnicity based on the PC1 and PC2 eigenvalues provided by the UK Biobank.
(TIF)
