# Supplementary material for: Identifying genetic variants associated with the ICD10 (International Classification of Diseases10)-based diagnosis of cerebrovascular disease using a large-scale biomedical database
Source: PLoS One. 2022 Aug 22;17(8):e0273217. doi: 10.1371/journal.pone.0273217 (PMC9394849; doi:10.1371/journal.pone.0273217)
Supplement: S1 Table — (DOCX) [file pone.0273217.s001.docx]

| Biometric/Biomarker | Cases  (N = 11,155) | Controls  (N = 122,705) | P Value | Clinical Significance |
| --- | --- | --- | --- | --- |
| Blood Type O (%) | 43% | 44% | <0.001 | N |
| Blood Type A (%) | 44% | 44% |  |  |
| Blood Type B (%) | 10% | 9% |  |  |
| Blood Type AB (%) | 4% | 4% |  |  |
| BMI (kg/m^2^) | 28.5 | 27.5 | <0.001 | N |
| Height (in) | 66.5 | 66.9 | <0.001 | N |
| Waist Circumference (in) | 37.6 | 36.4 | <0.001 | N |
| Weight (lbs) | 180 | 176 | <0.001 | N |
| Systolic blood pressure (mmHg) | 145 | 144 | <0.001 | N |
| Diastolic blood pressure (mmHg) | 83.0 | 82.9 | NS | N |
| Mean arterial blood pressure (mmHg) | 103 | 103 | <0.001 | N |
| Pulse Pressure (mmHg) | 60.3 | 59.1 | <0.001 | N |
| Pulse Rate (bpm) | 70.6 | 69.3 | <0.001 | N |
| Alkaline Phosphatase (IU/L) | 89.7 | 84.3 | <0.001 | N |
| Calcium (mg/dL) | 9.50 | 9.54 | <0.001 | N |
| Vitamin D (ng/mL) | 19 | 20 | <0.001 | N |
| Insulin-like growth factor (nmol/L) | 157 | 160 | <0.001 | N |
| Sex Hormone Binding Globulin (nmol/L) | 48.7 | 49.3 | <0.05 | N |
| Testosterone (ng/dL) | 232 | 235 | NS | N |
| Apolipoprotein A (mg/dL) | 148 | 153 | <0.001 | N |
| Apolipoprotein B (mg/dL) | 96.7 | 103 | <0.001 | N |
| Total Cholesterol (mg/dL) | 202 | 218 | <0.001 | M |
| C Reactive Protein (mg/dL) | 99.5 | 74.4 | <0.001 | M |
| HDL (mg/dL) | 51.8 | 54.9 | <0.001 | N |
| LDL (mg/dL) | 125 | 137 | <0.001 | M |
| Lipoprotein A (mg/dL) | 22.8 | 21.1 | <0.001 | N |
| Triglycerides (mg/dL) | 167 | 161 | <0.001 | N |
| Glucose (mg/dL) | 98.3 | 93.4 | <0.001 | N |
| Hemoglobin A1c (%) | 5.7 | 5.5 | <0.001 | N |
| Hemoglobin (g/dL) | 14.3 | 14.4 | <0.001 | N |
| Hematocrit (percent) | 41.5 | 41.8 | <0.001 | N |
| Mean Corpuscular Volume (fL) | 91.8 | 91.4 | <0.001 | N |
| Mean Corpuscular Hemoglobin (pg) | 31.7 | 31.6 | <0.05 | N |
| Mean Corpuscular Hemoglobin Concentration (g/dL) | 34.5 | 34.5 | <0.001 | N |
| White Blood Cell Count (1x 10^9^/L) | 7.4 | 6.9 | <0.001 | N |
| Platelet Count (1 x 10^9^/L) | 251 | 246 | <0.001 | N |
| Albumin (g/dL) | 4.46 | 4.51 | <0.001 | N |
| Alanine Transaminase (U/L) | 24.4 | 24.0 | <0.05 | N |
| Aspartate Transaminase (U/L) | 27.4 | 26.8 | <0.001 | N |
| Bilirubin — Total (mg/dL) | 0.55 | 0.57 | <0.001 | N |
| Bilirubin — Direct (mg/dL) | 0.116 | 0.113 | <0.001 | N |
| Gamma-glutamyl Transferase (U/L) | 47.6 | 39.0 | <0.001 | N |
| Blood Urea Nitrogen (mg/dL) | 35.2 | 33.8 | <0.001 | N |
| Creatinine (mg/dL) | 0.79 | 0.75 | <0.001 | N |
| Cystatin C (mg/L) | 1.02 | 0.94 | <0.001 | N |
| Phosphate (mg/dl) | 3.56 | 3.56 | <0.05 | N |
| Protein — Total (g/dL) | 7.23 | 7.23 | NS | N |
| Uric Acid (mg/dL) | 5.66 | 5.46 | <0.001 | N |
| Microalbumin – Urine (mg/L) | 59.6 | 31.0 | <0.001 | M |
| Creatinine – Urine (mmol/L) | 9710 | 9150 | <0.001 | L |
| Potassium — Urine (mEq/L) | 64.6 | 64.8 | NS | N |
| Sodium — Urine (mEq/L) | 77.5 | 77.6 | NS | N |

NS: Not significant, p > 0.05; N: No clinical significance; L: Low clinical significance; M: Medium clinical significance; High clinical significance
