# Supplementary material for: Identifying genetic variants associated with the ICD10 (International Classification of Diseases10)-based diagnosis of cerebrovascular disease using a large-scale biomedical database
Source: PLoS One. 2022 Aug 22;17(8):e0273217. doi: 10.1371/journal.pone.0273217 (PMC9394849; doi:10.1371/journal.pone.0273217)
Supplement: S2 Table — (DOCX) [file pone.0273217.s002.docx]

| ICD10 Diagnosis | Cases  (N = 11,155) | Controls  (N = 122,705) | P Value |
| --- | --- | --- | --- |
| Disorientation; unspecified (R41.0) | 6% | 1% | <0.001 |
| Malaise and fatigue (R53) | 5% | 1% | <0.001 |
| Tendency to fall; not elsewhere classified (R29.6) | 6% | 1% | <0.001 |
| Epilepsy; unspecified (G40.9) | 6% | 1% | <0.001 |
| Peripheral vascular disease; unspecified (I73.9) | 6% | 1% | <0.001 |
| Chronic renal failure (N18.9) | 6% | 1% | <0.001 |
| Pneumonia; unspecified (J18.9) | 6% | 1% | <0.001 |
| Left ventricular failure (I50.1) | 6% | 1% | <0.001 |
| Pleural effusion; not elsewhere classified (J90) | 6% | 1% | <0.001 |
| Dizziness and giddiness (R42) | 7% | 1% | <0.001 |
| Acute renal failure; unspecified (N17.9) | 10% | 2% | <0.001 |
| Lobar pneumonia; unspecified (J18.1) | 8% | 2% | <0.001 |
| Headache (R51) | 9% | 2% | <0.001 |
| Unstable angina (I20.0) | 6% | 2% | <0.001 |
| Dyspnea (R06.0) | 7% | 2% | <0.001 |
| Hyperlipidemia; unspecified (E78.5) | 8% | 2% | <0.001 |
| Dysphagia (R13) | 6% | 2% | <0.001 |
| Gastroenteritis and colitis of unspecified origin (A09.9) | 5% | 2% | <0.001 |
| Iron deficiency anemia; unspecified (D50.9) | 5% | 2% | <0.001 |
| Presence of aortocoronary bypass graft (Z95.1) | 7% | 2% | <0.001 |
| Personal history of diseases of the nervous system and sense organs (Z86.6) | 26% | 2% | <0.001 |
| Unspecified acute lower respiratory infection (J22) | 9% | 2% | <0.001 |
| Other forms of chronic ischemic heart disease (I25.8) | 9% | 2% | <0.001 |
| Procedure not carried out because of contraindication (Z53.0) | 6% | 2% | <0.001 |
| Alcohol use (Z72.1) | 7% | 2% | <0.001 |
| Arthrosis; unspecified (M19.9) | 6% | 2% | <0.001 |
| Other chest pain (R07.3) | 7% | 2% | <0.001 |
| Chronic obstructive pulmonary disease; unspecified (J44.9) | 10% | 3% | <0.001 |
| Tobacco use (Z72.0) | 11% | 3% | <0.001 |
| Presence of coronary angioplasty implant and graft (Z95.5) | 7% | 3% | <0.001 |
| Other specified abnormal findings of blood chemistry (R79.8) | 7% | 3% | <0.001 |
| Nausea and vomiting (R11) | 10% | 3% | <0.001 |
| Esophagitis (K20) | 5% | 3% | <0.001 |
| Depressive episode; unspecified (F32.9) | 11% | 3% | <0.001 |
| Other chemotherapy (Z51.2) | 6% | 3% | <0.001 |
| Syncope and collapse (R55) | 11% | 3% | <0.001 |
| Harmful use (F17.1) | 11% | 3% | <0.001 |
| Retention of urine (R33) | 7% | 3% | <0.001 |
| Constipation (K59.0) | 10% | 3% | <0.001 |
| Anemia; unspecified (D64.9) | 10% | 3% | <0.001 |
| Old myocardial infarction (I25.2) | 12% | 3% | <0.001 |
| Gastro-esophageal reflux disease with esophagitis (K21.0) | 5% | 3% | <0.001 |
| Hemorrhage of anus and rectum (K62.5) | 6% | 4% | <0.001 |
| Obesity; unspecified (E66.9) | 8% | 4% | <0.001 |
| Urinary tract infection; site not specified (N39.0) | 14% | 4% | <0.001 |
| Non-infective gastro-enteritis and colitis; unspecified (K52.9) | 9% | 4% | <0.001 |
| Unspecified hemorrhoids without complication (I84.9) | 5% | 4% | <0.001 |
| Hypothyroidism; unspecified (E03.9) | 8% | 4% | <0.001 |
| Unknown and unspecified causes of morbidity (R69) | 8% | 4% | <0.001 |
| Other and unspecified abdominal pain (R10.4) | 8% | 4% | <0.001 |
| Family history of ischemic heart disease and other diseases of the circulatory system (Z82.4) | 11% | 4% | <0.001 |
| Personal history of long-term (current) use of anticoagulants (Z92.1) | 20% | 4% | <0.001 |
| Presence of orthopedic joint implants (Z96.6) | 8% | 4% | <0.001 |
| Polyp of colon (K63.5) | 7% | 4% | <0.001 |
| Personal history of diseases of the circulatory system (Z86.7) | 47% | 4% | <0.001 |
| Personal history of allergy to penicillin (Z88.0) | 10% | 4% | <0.001 |
| Chronic ischemic heart disease; unspecified (I25.9) | 18% | 5% | <0.001 |
| Gastritis; unspecified (K29.7) | 9% | 5% | <0.001 |
| Procedure not carried out for other reasons (Z53.8) | 9% | 5% | <0.001 |
| Gastro-esophageal reflux disease without esophagitis (K21.9) | 10% | 5% | <0.001 |
| Unspecified hematuria (R31) | 9% | 5% | <0.001 |
| Personal history of diseases of the digestive system (Z87.1) | 10% | 5% | <0.001 |
| Atrial fibrillation and flutter (I48) | 23% | 6% | <0.001 |
| Gonarthrosis; unspecified (M17.9) | 7% | 6% | <0.001 |
| Angina pectoris; unspecified (I20.9) | 17% | 6% | <0.001 |
| Hyperplasia of prostate (N40) | 9% | 6% | <0.001 |
| Cataract; unspecified (H26.9) | 9% | 6% | <0.001 |
| Personal history of long-term (current) use of other medicaments (Z92.2) | 23% | 6% | <0.001 |
| Chest pain; unspecified (R07.4) | 17% | 7% | <0.001 |
| Non-insulin-dependent diabetes mellitus Without complications (E11.9) | 20% | 7% | <0.001 |
| Asthma; unspecified (J45.9) | 13% | 7% | <0.001 |
| Atherosclerotic heart disease (I25.1) | 18% | 7% | <0.001 |
| Diaphragmatic hernia without obstruction or gangrene (K44.9) | 14% | 9% | <0.001 |
| Diverticular disease of large intestine without perforation or abscess (K57.3) | 13% | 9% | <0.001 |
| Personal history of psychoactive substance abuse (Z86.4) | 30% | 10% | <0.001 |
| Pure hypercholesterolemia (E78.0) | 41% | 12% | <0.001 |
| Essential (primary) hypertension (I10) | 66% | 28% | <0.001 |
