# Supplementary material for: Identifying genetic variants associated with the ICD10 (International Classification of Diseases10)-based diagnosis of cerebrovascular disease using a large-scale biomedical database
Source: PLoS One. 2022 Aug 22;17(8):e0273217. doi: 10.1371/journal.pone.0273217 (PMC9394849; doi:10.1371/journal.pone.0273217)
Supplement: S3 Table — (DOCX) [file pone.0273217.s003.docx]

| SNP | Chromosome | Type of SNP | Gene | Mean Allele Frequency (%) | Odds Ratio | 95% Confidence Interval | P-value |
| --- | --- | --- | --- | --- | --- | --- | --- |
| rs61411276 | 4 | Intergenic | PITX2 | 20.2 | 1.11 | 1.07 – 1.15 | 2.59 x 10^-9^ |
| rs9685830 | 4 | Intergenic | PITX2 | 20.8 | 1.11 | 1.07 – 1.14 | 4.06 x 10^-9^ |
| rs1906615 | 4 | Intergenic | PITX2 | 20.1 | 1.11 | 1.07 – 1.14 | 4.10 x 10^-9^ |
| rs7440002 | 4 | Intergenic | PITX2 | 20.5 | 1.11 | 1.07 – 1.14 | 4.61 x 10^-9^ |
| rs6854111 | 4 | Intergenic | PITX2 | 20.1 | 1.11 | 1.07 – 1.14 | 5.00 x 10^-9^ |
| rs75259736 | 4 | Intergenic | PITX2 | 20.6 | 1.11 | 1.07 – 1.14 | 5.08 x 10^-9^ |
| rs2129982 | 4 | Intergenic | PITX2 | 20.2 | 1.11 | 1.07 – 1.14 | 5.27 x 10^-9^ |
| rs67249485 | 4 | Intergenic | PITX2 | 20.2 | 1.11 | 1.07 – 1.14 | 5.30 x 10^-9^ |
| rs13122916 | 4 | Intergenic | PITX2 | 20.6 | 1.11 | 1.07 – 1.14 | 5.33 x 10^-9^ |
| rs6847935 | 4 | Intergenic | PITX2 | 20.2 | 1.11 | 1.07 – 1.14 | 5.86 x 10^-9^ |
| rs9685833 | 4 | Intergenic | PITX2 | 20.7 | 1.11 | 1.07 – 1.14 | 6.22 x 10^-9^ |
| rs6837901 | 4 | Intergenic | PITX2 | 20.2 | 1.10 | 1.07 – 1.14 | 7.04 x 10^-9^ |
| rs7689774 | 4 | Intergenic | PITX2 | 20.2 | 1.10 | 1.07 – 1.14 | 7.06 x 10^-9^ |
| rs1906616 | 4 | Intergenic | PITX2 | 20.2 | 1.10 | 1.07 – 1.14 | 7.09 x 10^-9^ |
| rs6820568 | 4 | Intergenic | PITX2 | 20.2 | 1.10 | 1.07 – 1.14 | 7.45 x 10^-9^ |
| rs2466455 | 4 | Intergenic | PITX2 | 20.4 | 1.10 | 1.07 – 1.14 | 1.12 x 10^-8^ |
| rs6843082 | 4 | Intergenic | PITX2 | 20.2 | 1.10 | 1.07 – 1.14 | 1.14 x 10^-8^ |
| rs13143308 | 4 | Intergenic | PITX2 | 20.3 | 1.10 | 1.07 – 1.14 | 1.17 x 10^-8^ |
| rs2129983 | 4 | Intergenic | PITX2 | 20.5 | 1.10 | 1.07 – 1.14 | 1.27 x 10^-8^ |
| rs2634074 | 4 | Intergenic | PITX2 | 20.6 | 1.10 | 1.07 – 1.14 | 1.32 x 10^-8^ |
| rs2129977 | 4 | Intergenic | PITX2 | 20.2 | 1.10 | 1.07 – 1.14 | 1.48 x 10^-8^ |
| rs2723334 | 4 | Intergenic | PITX2 | 20.6 | 1.10 | 1.07 – 1.14 | 1.62 x 10^-8^ |
| rs1906599 | 4 | Intergenic | PITX2 | 20.5 | 1.10 | 1.06 – 1.14 | 1.80 x 10^-8^ |
| rs1922809 | 2 | Intronic | LRRTM4 | 42.7 | 1.08 | 1.05 – 1.11 | 4.29 x 10^-8^ |
