# Supplementary figures and images for: Identifying genetic variants associated with the ICD10 (International Classification of Diseases10)-based diagnosis of cerebrovascular disease using a large-scale biomedical database
Source: PLoS One. 2022 Aug 22;17(8):e0273217. doi: 10.1371/journal.pone.0273217 (PMC9394849; doi:10.1371/journal.pone.0273217)

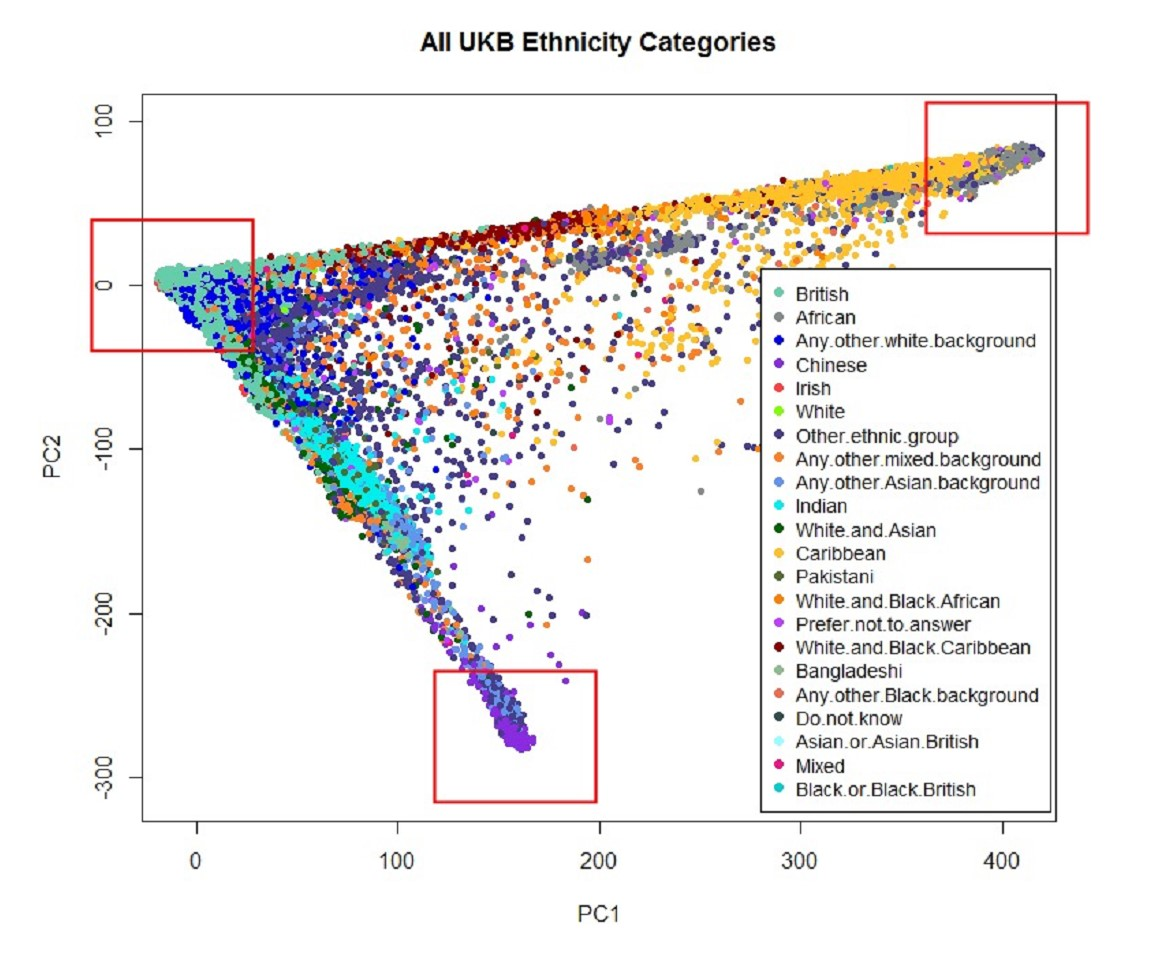

Supplement: S1 Fig — When selecting controls for comparison with cases, control subjects were selected from subjects within 80 units on the PC1 vs. PC2 graph. The size of 80 units is illustrated with the red boxes around subjects who are primarily European, Chinese, or African Ethnicity based on the PC1 and PC2 eigenvalues provided by the UK Biobank. (TIF) [file pone.0273217.s004.tif]
